# Role of Artificial Intelligence in Critical Care Medicine: A Literature Review

**DOI:** 10.7759/cureus.90149

**Published:** 2025-08-15

**Authors:** Ahmed M Abdelbaky, Wael G Elmasry, Ahmed H. Awad, Sarrosh Khan

**Affiliations:** 1 Critical Care Medicine, Mohammed Bin Rashid University of Medicine and Health Sciences (MBRU), Dubai, ARE; 2 Anaesthesiology, Mohammed Bin Rashid University of Medicine and Health Sciences (MBRU), Dubai, ARE; 3 Internal Medicine, Mohammed Bin Rashid University of Medicine and Health Sciences (MBRU), Dubai, ARE

**Keywords:** acute respiratory distress syndrome (ards), artificial intelligence (ai), critical care, icu (intensive care unit), sepsis

## Abstract

The growing availability of complex healthcare data, combined with advances in computational methods, has opened new avenues for improving critical care. Intensive care units (ICUs) generate continuous, multimodal data streams, ranging from vital‐sign waveforms to laboratory results and clinical notes that place a substantial cognitive burden on clinicians. In recent years, significant focus has emerged on the use of artificial intelligence (AI) in healthcare and the ICU. With an increase in interest and improvement in patient outcomes due to AI use in the ICU, there is a need for an updated summary of current evidence. This review highlights the growing promise of AI in several ICU domains. AI subdomains, machine learning (ML) and deep learning (DL) models, have been shown to accurately predict patient deterioration events such as sepsis, organ failure, and acute respiratory distress syndrome (ARDS) hours in advance. AI‐driven image interpretation can enhance diagnostic accuracy in radiology and pathology, and continuous monitoring algorithms can reduce false alarms. In conclusion, AI shows promise for critical care by enabling earlier risk detection, personalized therapy, and optimized resource utilization.

## Introduction and background

The growing burden of chronic diseases such as cardiovascular diseases, lung and colorectal cancers, chronic respiratory diseases like chronic obstructive pulmonary disease (COPD) and severe asthma, and the aging population has put significant strain on healthcare systems across the globe. However, modern advances in healthcare, primarily driven by the rapid growth of high-dimensional healthcare data and advancements in computational power, have transformed this sector [1]. This convergence has also provided unprecedented opportunities and redefined how we characterize health and disease as well as how we uncover the physiological and pathological mechanisms underlying critical illness. The application of these advancements to real-time clinical decision-making has also increased [2]. Among various sectors of healthcare, intensive care units (ICUs) demand real-time, precise data interpretation [3]. Critical care medicine, often delivered in ICUs, involves caring for patients with life-threatening conditions such as sepsis, respiratory failure, multiorgan dysfunction, severe trauma, and cardiac arrest who require continuous monitoring and complex interventions. ICU clinicians manage vast amounts of patient data that include continuous vital-sign waveforms, labs, and imaging. Therefore, healthcare professionals working in ICUs should make rapid, high-stakes decisions, as delays or errors can lead to severe harm or death [4,5].

The situation is further exacerbated as the ICU environment is highly heterogeneous and can include patients with trauma, sepsis, multiorgan failure, etc. The sheer volume and complexity of data in the ICU create a substantial cognitive burden on providers, thus a suitable candidate for advanced decision support tools [6]. Modern computerized decision support systems (CDSSs) are increasingly used in ICUs to improve care. Examples include automated alerts, protocol reminders, and risk scores. Studies report that well-designed clinical decision support (CDS) can improve guideline adherence and reduce medical errors in critical care [4]. However, so far, these tools have only modestly impacted patient outcomes. Experts, therefore, view artificial intelligence (AI) augmented support as the next step, one that might integrate diverse data streams such as vitals, labs, and notes into predictive, real-time guidance. Recent surveys involving Dutch (n = 64) and French (n = 171) ICU physicians have demonstrated an optimistic attitude among them. A large majority believe AI-based decision support could assist them in predicting readmission or mortality risk and improve patient care [7,8]. AI broadly refers to computational systems designed to mimic human cognitive functions such as learning, problem-solving, pattern recognition, and decision-making. Several subdomains of AI are being utilized in healthcare. For example, machine learning (ML) is a subset of AI where algorithms learn predictive models from data. Similarly, deep learning (DL) is an advanced form of ML using multilayer neural networks to model complex signals, and natural language processing (NLP) applies AI to interpret unstructured text such as clinical notes [9].

In healthcare, AI can analyze large, multidimensional data to recognize patterns and anticipate clinical events. For example, ML-driven tools, a field of AI, have already been applied to interpret medical images, monitor patients continuously, and flag early deterioration [10,11]. Across the globe, the transformative role of AI is being recognized by national health systems, research bodies, and regulatory agencies [9]. Although a considerable body of literature already exists on this topic, ongoing research continues to produce new findings and insights, highlighting the need for reproducibility, external validation, and real-world trials to ensure the reliability and applicability of these results. This creates a need for updated summaries of the evidence to better guide clinical practice. In particular, the role of AI in critical care medicine warrants focused attention due to its growing relevance and unique potential. This review aims to summarize current evidence on AI (ML and DL) applications in ICU care, focusing on early warning, diagnosis, treatment optimization, and workflow support.

## Review

Overview of AI in healthcare

Unique Challenges and Opportunities for AI in the ICU

AI techniques have been applied widely across various domains of healthcare. For example, DL has been used in medical imaging like in radiology and pathology to detect disease from images with high accuracy [12]. Studies report that AI-assisted mammogram analysis has reduced false positives/negatives, and convolutional neural networks (CNNs) have matched or exceeded dermatologists’ performance in identifying melanoma [13-15]. AI algorithms also excel at recognizing pneumonia on chest X-rays (CXRs) and spotting fractures on computed tomography (CT) scans, often faster and more consistently than human readers; however, their performance can vary significantly across different datasets and patient populations [16,17]. In laboratory medicine, ML is used to automate tasks like identifying pathogens or classifying blood smears, speeding up diagnoses. For instance, neural networks can classify bacteria from microscopic images or predict antibiotic resistance from genomic data [18]. Outside diagnostics, AI aids clinical workflows and decision-making. In emergency departments (EDs), for example, ML models have been developed to triage patients by analyzing vital signs and symptoms, thus prioritizing high-risk cases and optimizing patient flow [19]. AI-powered decision support can also provide real-time treatment suggestions and predict patient deterioration, potentially allowing earlier intervention.

The ICU is perhaps the most data-rich clinical setting. Patients are connected to monitors generating continuous streams of physiological data like electrocardiogram (ECG), pulse oximetry, and ventilator waveforms, on top of frequent lab tests and imaging. This abundance of data presents a major opportunity to apply advanced analytics and AI tools to enhance patient monitoring, predict clinical deterioration, and support timely interventions. In principle, this wealth of information can feed predictive models to forecast events like sepsis or organ failure hours before they occur [20]. However, leveraging ICU data also faces hurdles. Clinical data from the ICU can be highly heterogeneous and noisy. Furthermore, different devices and electronic health record (EHR) systems encode information differently, and missing or inconsistent entries are common [21]. Integrating these multisource data in real time remains technically challenging. Furthermore, clinicians may be reluctant to act on an opaque algorithm’s recommendation without understanding its reasoning. As noted by experts, biases in large healthcare databases and the lack of model interpretability make fairness and trust major issues [5]. Additional hurdles include legal and ethical barriers to sharing ICU data due to patient privacy regulations and the need for regulatory approval of AI tools [7].

Clinician attitudes further shape the landscape. Surveys of ICU physicians report generally positive expectations [7,8]. A survey of ICU physicians in two Dutch academic hospitals found that most were familiar with AI and viewed it positively [7]. About 86% believed AI could support their work, and 92% agreed that an AI-CDS tool could help guide discharge decisions, with site differences likely related to involvement in the tool’s development [7]. However, physicians emphasize that AI should augment rather than replace human judgment, especially given the complexity and stakes of ICU care. In ICUs, very few AI tools are widely used. Adoption has been slow due to workflow mismatch and unclear benefits. Even US Food and Drug Administration (FDA)-approved ICU AI devices have seen limited uptake [22]. Furthermore, ICU AI tools need extensive testing. Many published models are retrospective and not prospectively validated [22]. Another concern regarding the widespread use of AI in critical care is that it involves vulnerable patients. Issues like data privacy and accountability for AI-driven actions are amplified [5].

Applications of AI in critical care

AI in Early Warning Systems and Patient Monitoring

In an ICU, patient conditions can deteriorate rapidly; thus, timely recognition of clinical deterioration is vital. Traditional monitoring systems, while effective to an extent, often rely on threshold-based alerts that may miss subtle yet clinically significant trends. AI methods are increasingly used to predict patient deterioration hours before a crisis. For example, evidence shows that ML models using continuous ECG/heart rate variability (HRV) data have reached high accuracy for impending cardiac arrest. A DL model on 12-lead ECGs in 25,672 patients attained an area under the receiver operating characteristic curve (AUROC) of 0.948 for predicting in-hospital cardiac arrest within 24 hours [23]. Approaches using simpler signals like single-lead HRV also outperform standard warning scores [5,24]. ML algorithms are being developed to identify patients at risk of acute respiratory failure or needing intubation. Reviews highlight that predicting outcomes like the need for invasive ventilation and escalation of respiratory support are key targets, and ML has shown promise by automatically analyzing streams of vital signs and lab values to forecast respiratory decompensation [25].

Continuous real-time monitoring extends these early warnings [26]. AI-driven patient monitoring can fuse data from multiple sources like vitals and camera feeds and alert clinicians to pending crises. Real-time monitoring significantly improves patient safety by identifying hazards that are frequently overlooked in periodic checks. For instance, conditions like delirium can vary throughout the day, but sporadic evaluations often fail to detect these fluctuations [27]. Similarly, patients sometimes leave their beds without assistance, increasing their risk of falling, yet consistently tracking these moments as they happen is difficult. Advanced computer vision systems can offer instant, context-sensitive information about patient activities [28], staff-patient interactions, and environmental factors within the room [29]. These intelligent systems outperform conventional spot-checking methods by recognizing subtle, ongoing patterns that can support clinical decisions [30].

Equally important is alarm management. ICU monitors generate an overwhelming number of alerts, most of them false. For example, one report found 88.8% of arrhythmia alarms were false positives [31]. AI classifiers are being used to filter these out. By analyzing the raw waveform patterns and context, ML models can suppress spurious alerts while preserving true warnings. For instance, research demonstrated that a DL method could drastically reduce false arrhythmia alarms in the ICU without missing actual events [32].

Sepsis Prediction

Sepsis continues to pose a substantial challenge in ICUs and significantly influences patient prognosis, strains medical resources, and disrupts workflow efficiency. Current estimates suggest that nearly one-third of individuals admitted to ICUs are diagnosed with sepsis [33]. On a global scale, it impacts between 25 and 50 million people every year, ranking among the primary causes of death [34]. Detecting and treating sepsis remains particularly difficult due to its varied clinical manifestations and the highly individualized responses patients exhibit. Several tools, including rule-based systems, AI-based models, and traditional scoring systems, have been developed to assess sepsis risk and assist healthcare providers [35,36]. Traditional scoring tools such as the Sequential Organ Failure Assessment (SOFA) and Acute Physiology and Chronic Health Evaluation (APACHE II, III, IV) have been extensively used in critical care, but they have certain limitations. These models, introduced several decades ago, were constructed based on the clinical patterns and ICU populations of that era [37,38]. Since then, substantial shifts in patient profiles and healthcare practices have eroded their precision and relevance in the current sepsis landscape.

Recently, AI has been increasingly used to detect the risk of sepsis and its outcomes in ICU patients. Several ML models have demonstrated their efficacy in predicting the risk of sepsis and perform better than traditional scoring systems. For example, in their landmark trial, Adams et al. conducted a prospective multisite cohort study involving 6,877 sepsis patients detected by the Targeted Real-Time Early Warning System (TREWS) alert system prior to antibiotic initiation. Provider confirmation of the alert within three hours was linked to a 3.3% (95% CI: 1.7-5.1) adjusted absolute reduction and an 18.7% (95% CI: 9.4-27.0) adjusted relative reduction in in-hospital mortality, along with lower rates of organ failure and shorter hospital stays compared to delayed confirmations. In high-risk patients, the mortality benefit was even greater, showing a 4.5% (95% CI: 0.8-8.3) adjusted absolute reduction [36]. Liliopoulos et al. developed the VIOSync Sepsis Prediction Index using XGBoost, which outperformed qSOFA [39]. Similarly, another study on the Medical Information Mart for Intensive Care (MIMIC)-III dataset (5,800+ ICU admissions) compared five traditional ML methods and five DL architectures, specifically temporal convolutional networks (TCNs) and long short-term memory (LSTM) networks, among others, using the area under the precision-recall curve (AUPRC) as the metric. The DL models consistently outperformed both classical ML and conventional scoring tools for detecting sepsis more than four hours before onset [40]. Some reviews also report that AI algorithms can predict sepsis onset four to six hours earlier than clinical signs. For example, a meta-analysis by Islam et al. evaluated the performance of ML models in predicting sepsis and found that they outperformed traditional scoring systems. The pooled AUROC for ML models was 0.89 (95% CI: 0.86-0.92), indicating high predictive accuracy, compared to lower values for conventional tools like Systemic Inflammatory Response Syndrome (SIRS) (0.70) and Modified Early Warning Score (MEWS) (0.50). ML models also showed superior sensitivity (81%) and specificity (72%) in predicting sepsis onset three to four hours in advance [41]. However, Goh et al., based on an independent test sample, reported that their AI algorithm could predict sepsis 12 hours before the onset of sepsis [42].

Some studies have also used ML models for predicting sepsis-related mortality in the ICU. Gao et al., in their study, developed ML models to predict sepsis mortality with high accuracy using a carefully selected set of 38 clinical features from the MIMIC-IV database. Among the tested models, the Random Forest achieved the best performance with an AUROC of 0.94 (±0.01), outperforming other approaches [43]. Similarly, a systematic review by Mușat et al. analyzed 19 studies that developed ML models to predict survival in ICU patients with sepsis, using common clinical and laboratory data [44]. ML models, particularly gradient boosting decision trees, consistently outperformed traditional scoring systems like SOFA and APACHE, with the best model achieving an area under the curve (AUC) of 0.992, accuracy of 95.4%, and sensitivity of 91.7%; however, some studies had limited external validation. Frequently used predictors included age, albumin, lactate levels, and ventilator use [44]. Studies have also used AI to predict sepsis in real-world settings. For example, Boussina et al. investigated the impact of a DL model on sepsis outcomes in a real-world ED setting. Following the implementation of a nurse-facing alert system, there was a significant (1.9%) absolute reduction in in-hospital sepsis mortality. Patients also showed a 5% absolute increase in sepsis bundle compliance. Additionally, patients showed a meaningful reduction in organ dysfunction progression, reflected by a 4% decrease in 72-hour SOFA score changes after sepsis onset [45].

Similarly, Fleuren et al., in their systematic review and meta-analysis, evaluated 28 studies involving 130 ML models for real-time sepsis prediction across various hospital settings. Most models were developed in ICUs, with reported AUROC values ranging from 0.68 to 0.99, indicating variable but generally promising accuracy [46]. The use of ML models has also been associated with improvement in sepsis-related patient outcomes in limited real-world trials. For example, Burdick et al., in their study, reported that the ML algorithm was associated with significant improvements, including an approximate 40% reduction in in-hospital mortality and a 32% decrease in hospital length of stay. Additionally, a notable reduction of nearly 23% was observed in 30-day readmission rates [47]. Table 1 shows a summary of AI use in predicting sepsis and its outcomes.

**Table 1:** Use of AI for predicting sepsis and related outcomes ML: machine learning; MIMIC: Medical Information Mart for Intensive Care; ICU: intensive care unit; DL: deep learning; AI: artificial intelligence; AKI: acute kidney injury; CBC: complete blood count; AUROC: area under the receiver operating characteristic curve; qSOFA: quick Sequential Organ Failure Assessment

Author and year	Design	Study setting	Number of participants	AI application	Purpose of AI	External validation	Results	Accuracy	Precision	Sensitivity	AUROC
Liliopoulos et al. (2024) [39]	Retrospective study	Single-center real-world data	40,336	ML application	Sepsis prediction	No	ML model outperformed traditional tools like the qSOFA score	97%	NA	87%	0.98
Solís-García et al. (2023) [40]	Experimental study	Single-center real-world data	MIMIC-III database v1.4; 45,000	ML application	Sepsis prediction	No	Accurately predicted sepsis 4 hours before onset	95.6%	NA	NA	0.9491
Mușat et al. (2024) [44]	Systematic review	NA	19 studies	ML application	Sepsis outcome prediction	NA	Gradient boosting decision tree was the best model	95.4%	NA	91.7%	0.992
Wang et al. (2021) [48]	Observational cohort study	Single center	4,449	ML application	Sepsis prediction	No	Good predictive ability in sepsis patients, with specificity of 89%	NA	NA	87%	0.91
Gao et al. (2024) [43]	Retrospective study	Single-center real-world data	MIMIC-IV; over 40,000 ICU patients	ML models	Sepsis mortality	No	Random forest model was most effective	86.3%	87.6%	83.7%	0.94
Boussina et al. (2024) [45]	Quasi-experimental study	Multicenter	6,217	DL model	Sepsis prediction model on quality of care and survival	No	DL model resulted in 1.9% absolute reduction in in-hospital sepsis mortality; 5.0% increase in bundle compliance	NA	NA	80%	NA
Goh et al. (2021) [42]	Retrospective study	Single center	318	AI model	Early sepsis prediction	No	AI increased sepsis prediction by 32% and reduced false positives by 17%	NA	NA	87%	0.94
Burdick et al. (2020) [47]	Prospective study	Multicenter	75,147	ML algorithm	Sepsis-related mortality, length of stay, and readmission	No	39.5% reduction of in-hospital mortality, a 32.3% reduction in hospital length of stay, and a 22.7% reduction in 30-day readmission rate	NA	NA	NA	NA
Al-Ansari et al. (2025) [49]	Retrospective study	Multicenter	23,470	ML application	Sepsis-related mortality	No	Extra Trees model had the best performance, with specificity of 94.89%	90.9%	84.1%	94.8%	NA
Jiang et al. (2025) [50]	Retrospective study	Multicenter	46,097	ML model	Sepsis-associated AKI	Yes	The model is effective for identifying sepsis-associated AKI in the ICU	85.0%	69.2%	NA	0.932
Shen et al. (2024) [51]	Retrospective study	Multicenter	4,564	ML model	Sepsis-associated 28-day mortality	No	XGBoost algorithm showed best performance	74.2%	NA	70.3%	0.821
Steinbach et al. (2024) [52]	Retrospective study	Single center	1,488 sepsis cases	ML application on CBC	Sepsis in ICU	Yes	CBC-based ML can accurately predict sepsis in ICU	NA	NA	NA	0.872
Zhang et al. (2024) [53]	Retrospective study	Multicenter real-world data	3,535	ML application	Sepsis in-hospital mortality	No	XGBoost had the best performance of 7 ML models	NA	NA	NA	0.94

Acute Respiratory Distress Syndrome

Acute respiratory distress syndrome (ARDS) frequently arises as a complication among adults in general intensive care settings [54]. A global study from 2016, which included 459 ICUs across 50 nations, found that ARDS affected approximately 10% of critical care patients and was linked to a mortality rate surpassing 40% [54]. ARDS arises from a variety of triggers, which can be infectious or non-infectious. The development of ARDS is driven by complex biological mechanisms involving pulmonary inflammation, damage to both the lungs’ endothelial and epithelial linings, heightened vascular permeability, and disturbances in coagulation processes [55]. Early diagnosis of ARDS is crucial for its management. AI has shown potential to aid in the earlier diagnosis of ARDS, which may be detected late by clinicians. For example, a systematic review by Yang et al. showed that ML has good predictive performance for ARDS. The pooled AUROC was 0.74, with sensitivities and specificities of 67% and 68%, respectively [56]. Similarly, Pai et al. developed an AI-based diagnostic approach for ARDS by integrating clinical data and CXR analysis. The ensemble models combining CNN with ML algorithms achieved high performance, with AUC values ranging from 0.916 to 0.925. The model also identified the 15 most influential clinical features for ARDS detection and utilized Gradient-weighted Class Activation Mapping (Grad-CAM) to enhance interpretability [57].

Several other studies have demonstrated the usefulness of various AI, ML, and DL models in ARDS prediction. For ARDS, a recent review found that ML algorithms analyzing multimodal ICU data can identify early patterns of respiratory distress that signal ARDS, potentially enabling timelier diagnosis and intervention [58]. Similarly, a systematic review by Tran et al. reported that gradient boosting was the most commonly and effectively used ML method in ARDS prediction and management, applied in approximately 23% of the studies [59]. A study by Jiang et al. investigated the effectiveness of an ML model in predicting ARDS in ICU patients with sepsis using initial clinical data. Their findings showed that the Gaussian Naive Bayes algorithm was the best performer, achieving an AUC of 0.781, an accuracy of 78.6%, and a sensitivity of 82.4% in the test set. Ten key predictors were identified, with PaO_2_/PAO_2_ emerging as the most influential variable [60]. Some studies have also investigated ML models to predict mortality risk in ICU patients with ARDS. Using data from 5,732 patients, the XGBoost model demonstrated the best predictive performance with an AUROC of 0.887 and an AUPRC of 0.731. The model effectively identified high-risk patients early [61].

AI in Diagnosis and Prognostication

AI tools enhance diagnostic accuracy and outcome prediction in critical illnesses. By mining large EHR databases and images, ML models can recognize conditions like ARDS or acute kidney injury (AKI) that clinicians may otherwise detect late. AKI frequently develops in individuals admitted to ICUs and is recognized as an independent predictor of death among critically ill patients [62]. To date, there are no targeted therapies capable of fully reversing acute renal damage, and only a minority of those who survive regain complete kidney function [63]. In AKI, ML models trained on ICU data have shown strong potential to predict AKI onset and recommend management. For example, Mi et al. assessed the potential of ML models in predicting AKI within seven days of ICU admission. Among five ML models tested, the random forest model showed the best performance with an AUC of 0.81 and an accuracy of 0.79. In comparison, the traditional SOFA score achieved an AUC of 0.66 and an accuracy of 0.71 [64]. AI approaches can also forecast AKI development and severity. For instance, using high-frequency hemodynamic and lab data, ML models can predict which septic patients will develop severe AKI and even who will recover quickly after injury [65].

Similar efficacy of AI has been reported in other conditions. For example, a study by Zhang et al. reported that integrating HRV features with clinical parameters significantly improved ICU mortality prediction compared to traditional scoring systems like Acute Physiology Score (APS) III. The ensemble model achieved the highest predictive accuracy (AUROC = 0.878), with XGB also performing well (AUROC = 0.869), both outperforming APS III (AUROC = 0.765) [66]. A significant number of studies have built ML risk scores for ICU mortality and length of stay [67,68]. For example, one large study trained random forest models on >12,000 ICU admissions and achieved an AUC of 0.945 for mortality prediction and an AUC of 0.88 for classifying short vs. long ICU stays [67]. Similarly, a study by Mao et al. developed an ML-based model to predict in-hospital mortality in ICU patients with spontaneous intracerebral hemorrhage (sICH). Among five models tested, XGBoost showed the best performance with an AUC of 0.907 in internal validation and 0.787 in external validation, showing that the XGBoost-based tool can effectively support early mortality risk assessment in sICH patients [68].

AI in Decision Support and Treatment Optimization

AI is also being applied to guide therapies and personalize ICU treatment protocols. Examples include ventilator management, sedation titration, and fluid/antimicrobial strategies. ML models have been developed to help manage mechanical ventilation. Studies have used patient signals to predict weaning readiness and tailor ventilator settings. For example, a review noted that AI has been tested for tasks like predicting successful extubation and optimizing respiratory parameters [69]. Similarly, a study by Liao et al. developed an AI model to predict the optimal timing for weaning patients from prolonged mechanical ventilation in a respiratory care center, using data from 670 patients. All seven models demonstrated strong performance, with AUC values ranging from 0.792 to 0.868. Implementation of the best model through an interactive dashboard resulted in a reduction of 0.5 ventilator days per patient [70]. Liu et al. also supported these findings, showing that a two-stage AI prediction model can predict timing to wean intubated patients in the ICU [71]. Several other studies have investigated AI use in mechanical ventilation and show promising results [72].

AI has shown promise in individualizing fluid resuscitation strategies for ICU patients. For example, an ML-derived policy tree identified which septic-AKI patients would benefit from a restrictive fluid strategy (defined as <500 mL in 24 hours). In validation, patients flagged for restrictive fluids by AI had much higher AKI reversal rates and fewer adverse kidney outcomes than those who received liberal fluids [65]. Another potential application of AI in critical care is optimization of antibiotic use. AI-CDSS tools can recommend empiric antibiotic choices or dosing by analyzing patient data and microbiology patterns, learning from historical cases to suggest narrow-spectrum therapy when appropriate and flagging mismatches in prescribed antibiotics [73,74]. A meta-analysis of 27 studies found that ML models significantly outperformed traditional methods in antimicrobial stewardship for sensitivity (p = 0.009) and negative predictive value (p < 0.001) [75].

Workflow and Imaging Data Integration

AI’s role in the ICU extends beyond individual patients to operational efficiency. Predictive models use trends in admissions, discharges, and census to forecast ICU bed availability. In practice, AI systems can display real-time bed status and near-future forecasts [76,77]. AI-driven scheduling tools match nursing and physician staffing to projected patient load. Algorithms are also being developed to prioritize ICU admissions based on severity scores and to distribute tasks such as medication orders, diagnostic testing, and routine monitoring. While most published AI triage systems focus on the ED, similar approaches can assign ICU-level resources [19,78]. AI can also optimize the use of equipment and space. For example, AI can analyze OR scheduling data to suggest surgery timings that balance ICU bed needs [1]. In supply chain management, AI can forecast inventory usage such as ventilator circuits and IV fluids during surges and automate orders, which can ensure that necessary supplies are pre-stocked for ICU demands.

Imaging analysis in the ICU is another fruitful AI application. DL can interpret radiographs, CT scans, and ultrasound at the bedside, often as an assistant to busy clinicians. For instance, AI has been applied to CXRs to distinguish causes of pneumonia. In one study of emergency ICU patients, an AI model achieved 97.5% sensitivity and 93.8% overall accuracy in differentiating coronavirus disease (COVID)-19 vs. bacterial pneumonia on portable CXRs [79]. Such AI tools can highlight subtle lung opacities or quantify infiltrates, aiding rapid diagnosis during pandemic surges or routine ARDS workup. Furthermore, AI guidance has revolutionized point-of-care ultrasound. Baloescu et al. in their study showed that with AI assistance, non-experts such as nurses and respiratory therapists acquired lung ultrasound images of expert-level quality 98.3% of the time [80]. AI is enabling multimodal data integration. AI can synthesize streams of continuous data such as vital signs, lab trends, waves of ventilator pressure, bedside images, and even clinician notes into an integrated assessment. For example, recent literature on critical care emphasizes that ML models can ingest vast, heterogeneous ICU data to improve predictions [25]. Table 2 shows the summary of key studies that have assessed AI use in critical care.

**Table 2:** Summary of key studies assessing AI use in critical care ML: machine learning; APS: Acute Physiology Score; MIMIC: Medical Information Mart for Intensive Care; ICU: intensive care unit; DL: deep learning; AI: artificial intelligence; AKI: acute kidney injury; AUROC: area under the receiver operating characteristic curve; AUPRC: area under the precision-recall curve; ARDS: acute respiratory distress syndrome; LOS: length of stay; TEE: transesophageal echocardiography; LV: left ventricular

Author and year	Design	Study setting	Number of participants	AI application	Purpose of AI	External validation	Results	Accuracy	Precision	Sensitivity	AUROC
Alparslan et al. (2025) [81]	Retrospective case control	Single center	289 (159 carbapenem-resistant and 130 carbapenem-susceptible)	ML (XGBoost algorithm)	Carbapenem-resistant *Klebsiella pneumoniae* infection in the ICUs	No	This AI model achieved an F1 score of 85% and a Matthews correlation coefficient of 0.66	83%	83%	88%	NA
Li et al. (2025) [82]	Retrospective study	Single-center real-world data	913	ML application	28-day all-cause mortality	No	Neural network algorithm showed the best performance	NA	NA	80%	0.801
Huang et al. (2025) [83]	Retrospective study	Single-center real-world data	2,838	ML application	Mortality due to short-term heart rate variability in ICU	No	Ensemble model had performance better than APS III	NA	NA	NA	0.878
Kim et al. (2024) [84]	Retrospective study	Single center	84 delirium patients, 336 non-delirium patients	ML application	To predict delirium in acute stroke patients in the ICU	No	ML model can predict real-time risk of delirium	42%	NA	NA	0.80
Lim et al. (2025) [85]	Retrospective study	Multicenter real-world data	Medical Information Mart for Intensive Care (MIMIC)-III and the eICU Collaborative Research Database (eICU-CRD)	ML; ensemble model	Predict ICU readmission within 48 hours after discharge	Yes	iREAD demonstrated better performance compared to traditional scores	NA	NA	NA	0.768 (0.748–0.787) and 0.725 (0.712–0.739) for overall readmission in MIMIC-III and eICU-CRD, respectively
Zaidat et al. (2025) [86]	Retrospective study	Single center	535	ML model	To predict prolonged ICU stay in adult spinal deformity patients	No	Random forest model had best performance	NA	NA	NA	0.83
Zhang et al. (2025) [66]	Retrospective study	Single-center real-world data	18,186 patients from MIMIC-IV v2.2 and MIMIC-III databases	ML model	To predict AKI in ICU	Yes	XGBoost had best performance	81.2%	NA	57.1%	0.88
Pan et al. (2025) [87]	Retrospective study	Multicenter	575	5 ML models	Mortality prediction in patients with severe community-acquired pneumonia	Yes	LightGBM model had best performance, with F1 score of 0.588	74.5%	43.9%	89.3%	0.842
Thiele et al. (2025) [88]	Retrospective study	Single center	7,156	ML models	High-risk patients in ICU	No	Artificial neural network was best able to predict deterioration, 10 h before documentation in clinical records	NA	NA	85%	0.81
Yang et al. (2025) [56]	Systematic review	NA	17 studies	ML models	ARDS prediction	NA	Diagnostic odds ratio was 6.26 whereas the positive likelihood ratio was 2.80	NA	NA	67%	0.740
Jiang et al. (2024) [60]	Retrospective study	Single center	279	ML model	ARDS prediction in sepsis patients	No	The model accurately predicted ARDS, with F1 score of 0.824	78.6%	NA	82.4%	0.781
Li et al. (2025) [61]	Retrospective study	Multicenter real-world data	5,732	Eight ML algorithms to construct predictive models	Mortality risk in ARDS patients	No	XGBoost model demonstrated the best predictive performance with an AUROC of 0.887 and an AUPRC of 0.731	NA	NA	NA	0.887 (95% CI: 0.863–0.909)
Yu et al. (2024) [89]	Prospective observational study	Single center	50	AI-based automated analysis of TEE	Real-time, continuous, and objective monitoring of LV function in critically ill postoperative patients	No	AI-enabled TEE monitoring demonstrated feasibility and reliability in continuous LV function assessment. It reduces operator dependency and enables earlier detection of cardiac dysfunction	NA	NA	NA	NA
Mao et al. (2024) [68]	Retrospective study	Multicenter real-world data	1,596	5 ML models	In-hospital mortality in patients with spontaneous intracerebral hemorrhage	Yes	XGBoost model showed best performance	87.4%	NA	58.2%	0.907
Liu et al. (2022) [71]	Retrospective study using a two-stage approach	Single center	10,045 cases	Two-stage AI model	To predict optimal weaning time and reduce ventilation time	No	Shorter mean ventilation time (144.3 h vs. 158.7 h). Shorter hospital LOS (22.2 d vs. 25.7 d). Weaning failure rate was 25.4%	NA	NA	NA	NA

Multimodal Large Language Models in Critical Care

Recent advances have seen the rapid emergence of foundation models and multimodal large language models (LLMs) tailored for healthcare applications, including critical care [90]. Unlike traditional task-specific ML systems, foundation models are pre-trained on massive, diverse datasets and can be adapted to multiple downstream tasks with minimal additional training. In the ICU setting, multimodal LLMs are being explored for real-time EHR summarization, integration of structured clinical data with unstructured text (e.g., clinician notes), waveform analysis, and even imaging interpretation [91]. These models can generate context-aware decision support recommendations, draft progress notes, and highlight pertinent clinical trends across time, potentially reducing documentation burden and improving situational awareness. Early studies suggest they can complement clinician judgment, but concerns remain regarding bias, hallucinations, interpretability, and the need for rigorous, prospective, multicenter validation in high-stakes ICU environments [92,93]. As such, the safe deployment of foundation and multimodal LLMs in critical care will require close collaboration between clinicians, data scientists, and regulatory bodies, along with robust evaluation frameworks.

Strengths and limitations

There are certain strengths and limitations of this review that should be acknowledged. The main strength of this review is that it summarizes the latest evidence, which was not included in previous systematic reviews. The majority of included studies were published in 2025. However, despite this strength, there are several limitations. Firstly, a systematic search was not conducted in key databases such as Web of Science or Scopus, which could have resulted in the omission of certain studies. Furthermore, we did not follow Preferred Reporting Items for Systematic Reviews and Meta-Analyses (PRISMA) reporting standards or present a reproducible search strategy and PRISMA flow diagram, making study identification and selection less transparent. There was no pre-registered protocol or explicit, reproducible inclusion/exclusion criteria, increasing the risk of selective citation and reviewer bias. We also did not perform a formal risk-of-bias or quality appraisal. Our search was conducted primarily in PubMed and Google Scholar for the identification of studies. Additionally, we searched the reference lists of relevant articles to identify further studies for inclusion. No quantitative synthesis was attempted; therefore, pooled effect estimates and measures of heterogeneity are unavailable. The search strategy also likely did not comprehensively cover gray literature or non-English sources, which could introduce publication and language bias. Finally, synthesis relied on qualitative interpretation, which, while useful for breadth, is inherently more subjective and less reproducible than systematic methods. Future work should consider a registered, PRISMA-compliant systematic review with formal quality assessment to address these limitations.

## Conclusions

AI shows strong promise in the ICU for early prediction, improved diagnosis, and workflow optimization. ML and DL models can leverage complex, multimodal ICU data to anticipate clinical deterioration, enhance diagnostic precision, and support more efficient care delivery. However, most current models are derived from retrospective, single-center datasets and lack robust real-world validation, limiting immediate clinical applicability. Future research should focus on prospective, multicenter trials, clearer regulatory frameworks, and the development of explainable AI approaches to ensure safe, transparent, and effective integration of AI tools into critical care practice.
